# l-Serine Lowers the Inflammatory Responses during Pasteurella multocida Infection

**DOI:** 10.1128/IAI.00677-19

**Published:** 2019-11-18

**Authors:** Fang He, Zheng Yin, Chenlu Wu, Yaoyao Xia, Miaomiao Wu, Pan Li, Huihui Zhang, Yuanyuan Yin, Nengzhang Li, Guoqiang Zhu, Wenkai Ren, Yuanyi Peng

**Affiliations:** a College of Animal Science and Technology, Southwest University, Chongqing, China; b Guangdong Provincial Key Laboratory of Animal Nutrition Control, Institute of Subtropical Animal Nutrition and Feed, College of Animal Science, South China Agricultural University, Guangzhou, China; c Jinan First People’s Hospital, Shandong, China; d Joint International Research Laboratory of Agriculture and Agri-Product Safety of Ministry of Education of China, Jiangsu Co-innovation Center for Important Animal Infectious Diseases and Zoonoses, College of Veterinary Medicine, Yangzhou University, Yangzhou, China; Washington State University

**Keywords:** inflammation, l-serine, macrophage, neutrophil, *Pasteurella multocida*

## Abstract

Pasteurella multocida causes a variety of infectious diseases in various species of mammals and birds, resulting in enormous economic loss to the modern livestock and poultry industry. However, the mechanism of host-pathogen interaction is unclear. Here, we found that l-serine levels were significantly decreased in murine lungs infected with P. multocida. Exogenous l-serine supplementation significantly increased the survival rate of mice and decreased the colonization of P. multocida in the lungs of mice. Notably, l-serine decreased the macrophage- and neutrophil-mediated inflammatory responses in mice during P. multocida infection.

## Abstract (precis)

Pasteurella multocida causes a variety of infectious diseases in various species of mammals and birds, resulting in enormous economic loss to the modern livestock and poultry industry. However, the mechanism of host-pathogen interaction is unclear. Here, we found that l-serine levels were significantly decreased in murine lungs infected with P. multocida.

## INTRODUCTION

Pasteurella multocida is a Gram-negative bacterium and primarily causes hemorrhagic septicemia and pulmonary inflammation in poultry and livestock (1–5). P. multocida is divided into five different serotypes, A, B, D, E, and F, based on different capsular antigens (6, 7). P. multocida serotype A always triggers bovine respiratory diseases with a high morbidity, mainly resulting in significant bovine pulmonary tissue lesions (8–11). Unfortunately, the pathogenesis and the host-pathogen interaction remain to be fully understood, and no effective methods are available to prevent and/or treat P. multocida infection.

Increasing studies have found that glucose, fatty acid, and amino acid metabolism play important roles in the pathogenesis of P. multocida (12–16). Notably, our previous investigations have shown that amino acids shape the pathogenesis of P. multocida infection. For example, supplementation with glutamine has beneficial effects against P. multocida infection in mice that were preimmunized with the inactivated P. multocida vaccine by enhancing general defense responses and decreasing expression of specific virulence factors (17). Furthermore, dietary proline or arginine supplementation enhances immune responses through increasing serum antibody titer and glutathione peroxidase (GSH-PX) level and decreasing the production of cytokines (e.g., interleukin-6 [IL-6], IL-8, and tumor necrosis factor alpha [TNF-α]) in inactivated P. multocida vaccine-immunized mice (18, 19). Based on the above investigations, we speculated that amino acid metabolism plays important roles in the pathogenesis of P. multocida infection. Therefore, this study was conducted to explore the interaction between host and P. multocida from the perspective of the amino acid metabolism.

Multiple lines of investigations have discovered a link between serine and immune cell function and even infection (20). For example, serine metabolism shapes the fate decision of immune cells, like T cells and macrophages, though one-carbon metabolism (21, 22) and glutathione (GSH) synthesis (23). However, the role of serine in immune responses during P. multocida infection is unknown. Here, we found that P. multocida infection remarkably shapes serine metabolism in the mouse lung. Notably, exogenous l-serine administration lowers bacterial colonization and macrophage- and neutrophil-mediated inflammation as well as enhances the survival rate in mice during P. multocida infection.

## RESULTS

### Serine metabolism changes during P. multocida infection.

**FIG 1:**
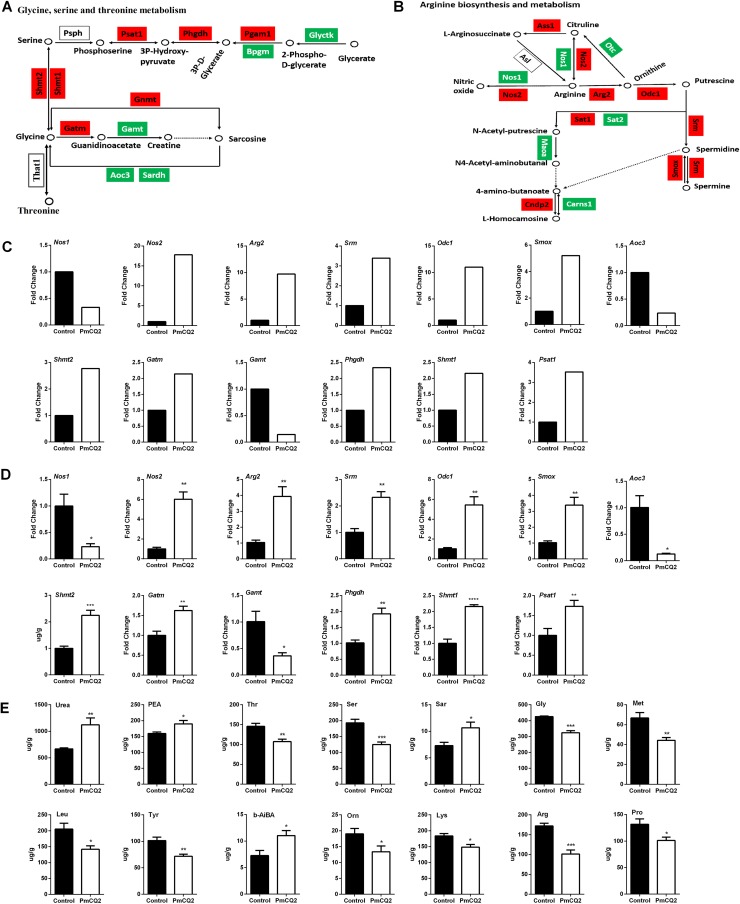
Amino acid metabolism change during P. multocida infection. Mice were infected with P. multocida by intraperitoneal injection, and the lung tissues were collected at 16 h after bacterial infection. (A) Alteration of glycine, serine, and threonine metabolism after P. multocida infection (*n* = 3). (B) Alteration of arginine metabolism after P. multocida infection (*n* = 3). (C) The expression of genes encoding the enzymes involved in the metabolism of glycine, serine, and threonine metabolism and arginine metabolism by RNA-seq (*n* = 3). (D) The expression of genes encoding the enzymes shown in panel C analyzed by qRT-PCR (*n* = 6). (E) The levels of amino acids in the lung tissues of mice infected by P. multocida (*n* = 6). The data were analyzed with unpaired *t* tests. (A and B) Genes in red boxes are upregulated, while those in green boxes are downregulated. Panels D and E were expressed as means ± SEM. *, *P* < 0.05; **, *P* < 0.01; ***, *P* < 0.001; ****, *P* < 0.0001.

Our previous study explored the differentially expressed genes (DEGs) during P. multocida infection in mice (9). Transcriptomic analysis identified DEGs from 16 amino acid biosynthesis pathways (see Table S1 in the supplemental material). Further analysis of these pathways suggested that l-serine, glycine and threonine metabolism (path:mmu00260) (Fig. 1A) and arginine biosynthesis and metabolism (path:mmu00330) (Fig. 1B) were obviously enriched. The changes of DEGs in path:mmu00260 and path:mmu00330 from transcriptomic analysis (Fig. 1C) were also validated by quantitative real-time PCR (qRT-PCR) at 16 h postinfection (Fig. 1D), although there was no significant difference at 4 h (see Fig. S1A in the supplemental material) and 8 h (Fig. S1B) postinfection. Notably, P. multocida infection enhanced the expression of *Psat1*, *Phgdh*, *Pgam1*, *Shmt1*, *Shmt2*, *Gnmt*, and *Gatm* (Fig. 1A, C, and D), suggesting that P. multocida infection promotes l-serine and glycine metabolism. In order to further validate the changes in amino acids during P. multocida infection, we determined the concentrations of free amino acids in the mouse lung using an L-8900 amino acid analyzer. The levels of 14 amino acids showed significant differences after infection; of these, 10 amino acids decreased (Ser, Gly, Thr, Arg, Pro, Tyr, Met, Leu, Lys, and Orn) (Fig. 1E). Collectively, these results indicate that P. multocida infection induces significant changes in amino acid metabolism, especially in l-serine, glycine, and threonine metabolism.

### Exogenous l-serine supplementation enhances resistance to P. multocida infection.

To further explore the potential effects of l-serine on mice infected with P. multocida, we supplemented mice with l-serine before infection. We supplemented the mice with l-serine through an intramuscular injection of 0.2 mg/kg of body weight. l-serine supplementation lowered bacterial colonization in the lungs, TNF-α level in the lungs, and IL-1β and gamma interferon (IFN-γ) levels in the serum during P. multocida infection (see Fig. S2 in the supplemental material). We then supplemented l-serine before infection through intranasal administration of serine with a dosage of 0.2 mg/kg because a previous study found that the drug is more effective when it is administered directly to lung tissue through trachea injection than by other methods (24). Serine lowered the bacterial colonization in the lungs and inflammatory cytokine production at 8 h and 16 h postinfection (see Fig. S3 in the supplemental material).

**FIG 2:**
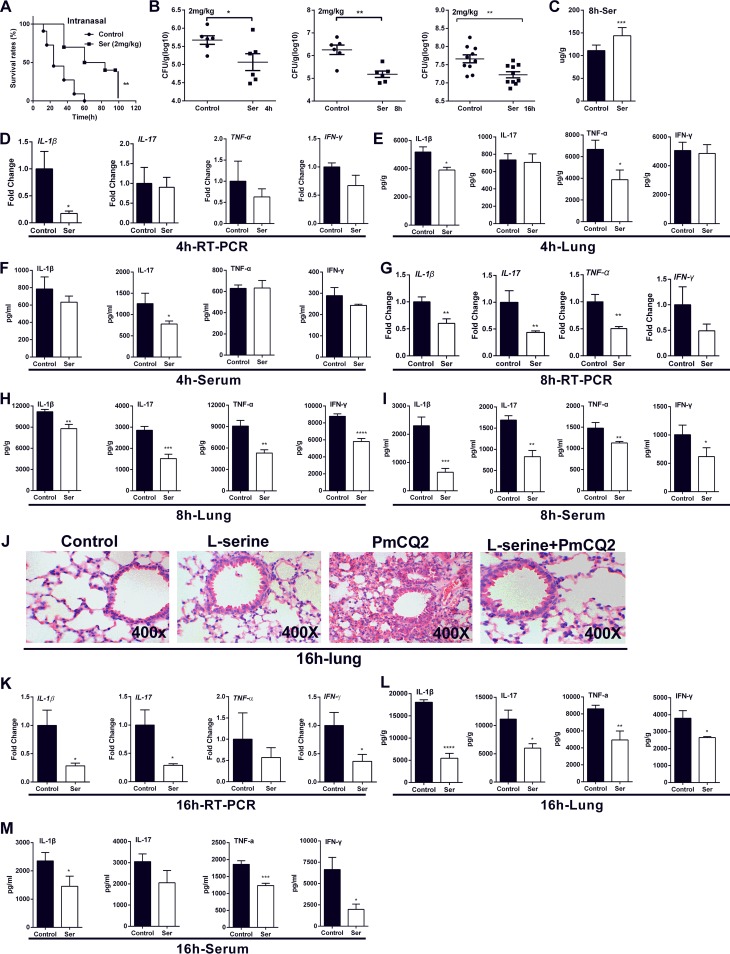
Exogenous l-serine supplementation enhances resistance to P. multocida infection. (A) l-serine improves the survival rate of mice (*n* = 10). (B) l-serine markedly decreases bacterial burden in mouse lungs infected by P. multocida at 4, 8, and 16 h postinfection (*n* = 6 or 10). (C) l-serine supplementation significantly increases the levels of l-serine in the lung tissues of mice at 8 h postinfection by P. multocida (*n* = 10). (D to F) Effect of l-serine on the mRNA expression and protein abundance of IL-1β, IL-17, IFN-γ, and TNF-α in the lung and serum at 4 h postinfection by P. multocida (*n* = 8 to 10). (G to I) Effect of l-serine on the mRNA expression and protein abundance of IL-1β, IL-17, IFN-γ, and TNF-α in the lung and serum at 8 h postinfection by P. multocida (*n* = 8 to 10). (J) H&E staining to analyze the inflammation in the mouse lung at 16 h postinfection by P. multocida (*n* = 8). (K to M) Effect of l-serine on the mRNA expression and protein abundance of IL-1β, IL-17, IFN-γ, and TNF-α in the lung and serum at 16 h postinfection by P. multocida (*n* = 8 to 10). Data in panels A, B, C, and J were pooled from three independent experiments (*n* = 6 to 10 total mice per group). Panels D to I and K to M are representative of two independent experiments with 8 to 10 replicates in each time. (D, E, G, H, K, and L) Total lung tissues were collected. Half of them were for RT-PCR analysis, and half were for ELISA. Data were analyzed by unpaired *t* test or Mann-Whitney test and expressed as means ± SEM. PmCQ2, P. multocida. *, *P* < 0.05; **, *P* < 0.01; ***, *P* < 0.001; ****, *P* < 0.0001.

Notably, the survival rate of mice infected with P. multocida was significantly increased by intranasal administration with 2 mg/kg l-serine (Fig. 2A). The bacterial colonization in the lung was decreased at 4 h, 8 h, and 16 h postinfection (Fig. 2B). Intranasal administration of serine significantly increased the lung levels of free l-serine, but not other amino acids, at 8 h postinfection (Fig. 2C; see Fig. S4A in the supplemental material). Serine supplementation inhibited the mRNA expressions and secretion of IL-1β, IL-17, IFN-γ, and TNF-α in the lungs and serum at 4 h (Fig. 2D to F), 8 h (Fig. 2G to I) and 16 h (Fig. 2K to M) postinfection, with the most significant changes at 8 and 16 h postinfection. Hematoxylin and eosin (H&E) staining also showed that intranasal administration of l-serine alleviated infection-induced pneumonia at 8 h postinfection (Fig. 2J). Interestingly, intranasal administration of serine did not change the levels of serine in the lung at 16 h postinfection (see Fig. S4B in the supplemental material). The possible reason may be that serine supplementation significantly increased expressions of l-serine metabolism-related enzymes, including *Shmt1* and *Phgdh*, at 16 h postinfection but not at 4 and 8 h postinfection (see Fig. S5 in the supplemental material).

The above data indicated that l-serine reduces the bacterial load of P. multocida and the inflammatory responses during infection. In order to explore whether these effects depend on direct inhibition of P. multocida growth, the influence of l-serine (even glycine and threonine) on the growth of P. multocida *in vitro* was explored. Different concentrations of l-serine, glycine, and threonine were added to culture medium for P. multocida. The results showed that different concentrations of l-serine, glycine, and threonine did not directly affect the growth of P. multocida in Martin broth medium or even in a nutrition-deficient LB medium (see Fig. S6A to C in the supplemental material). Also, l-serine, glycine, and threonine did not affect the phagocytosis of P. multocida by macrophages or its adhesion properties to macrophages and epithelial cells (see Fig. S6D and E in the supplemental material).

Collectively, these results imply that l-serine shows beneficial effects during P. multocida infection, including lower death rate, bacterial load, and inflammation in the lung.

### l-serine inhibits macrophage- and neutrophil-mediated inflammatory responses.

**FIG 3:**
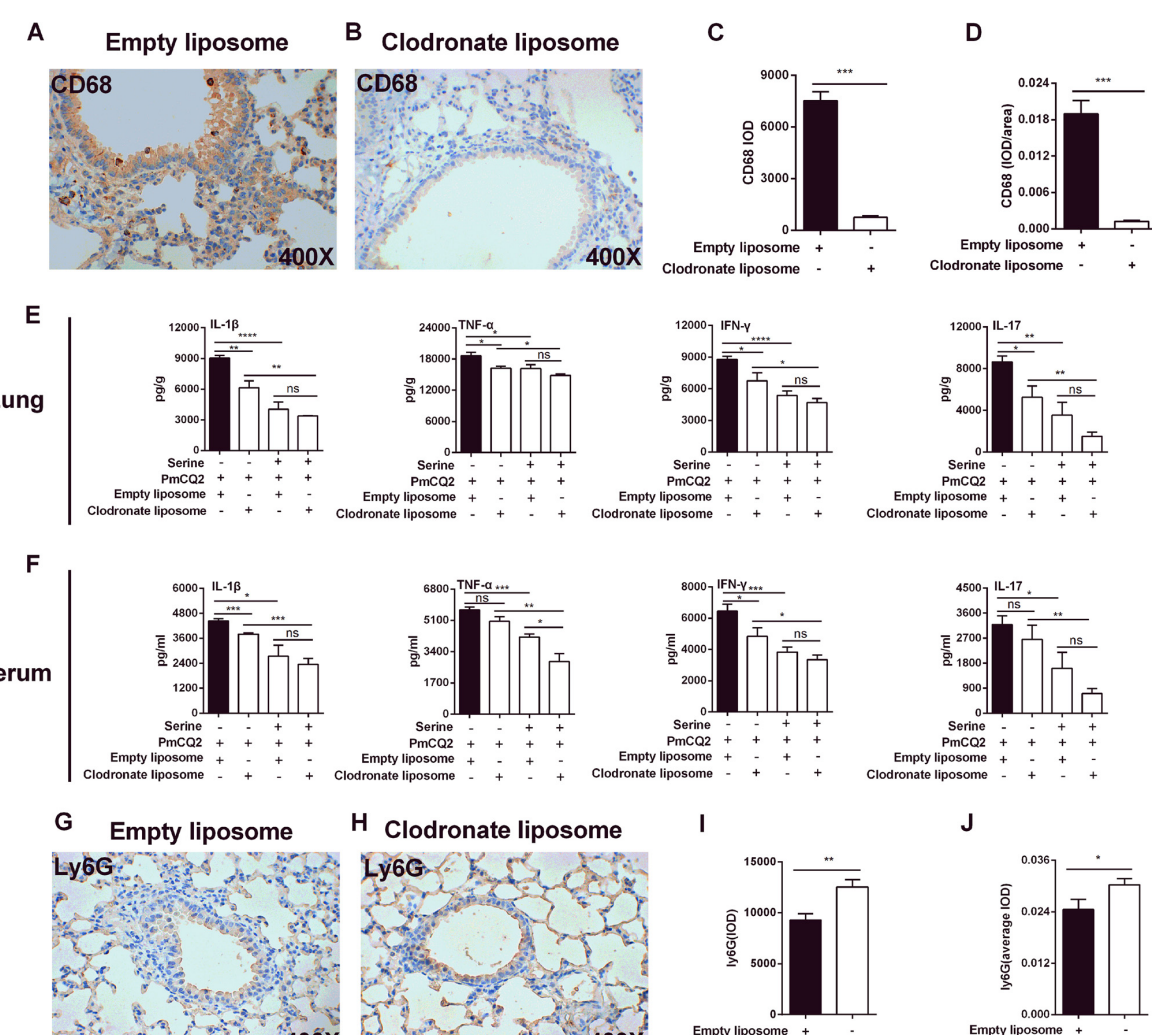
The role of l-serine in an alveolar macrophage-cleared mouse model of P. multocida infection. Mice were infected with P. multocida by intraperitoneal injection, and the lung tissues were collected at 16 h after bacterial infection. (A to D) The expression of CD68 was determined by immunohistochemistry (*n* = 8). (E and F) The production of IL-1β, IL-17, IFN-γ, and TNF-α in the lung and serum at 16 h after P. multocida infection (*n* = 6). (G to J) The expression of Ly6G was determined by immunohistochemistry (*n* = 8). The data in panels C, D, I, and J were determined by unpaired *t* test or Mann-Whitney test, and panels E and F were analyzed by one-way ANOVA. All data were expressed as means ± SEM. *, *P* < 0.05; **, *P* < 0.01; ***, *P* < 0.001; ****, *P* < 0.0001.

To determine whether l-serine administration inhibits host inflammatory responses by regulating the functions of alveolar macrophages, the alveolar macrophages *in vivo* were deleted using clodronate-loaded liposome. The deletion of alveolar macrophages was confirmed by immunoblotting for CD68 (Fig. 3A to D). In this model, serine supplementation decreased the secretion of inflammatory cytokines, including IL-1β, IL-17, IFN-γ, and TNF-α, both in the lung (Fig. 3E) and in serum (Fig. 3F) during P. multocida infection. The possible reason is that serine has no effect on alveolar macrophage-mediated inflammation during P. multocida infection, or there is a compensatory increase in neutrophils in this model. Interestingly, there was no change in the secretion of IL-1β, IL-17, IFN-γ, and TNF-α in the lung (Fig. 3E) or IL-1β, IL-17, and IFN-γ in the serum (Fig. 3F) after alveolar macrophage clearance, suggesting a compensatory increase in these cytokine-producing cells in the lung after macrophage clearance. Notably, there were significantly increased numbers of neutrophils after macrophage deletion (Fig. 3G to J).

**FIG 4:**
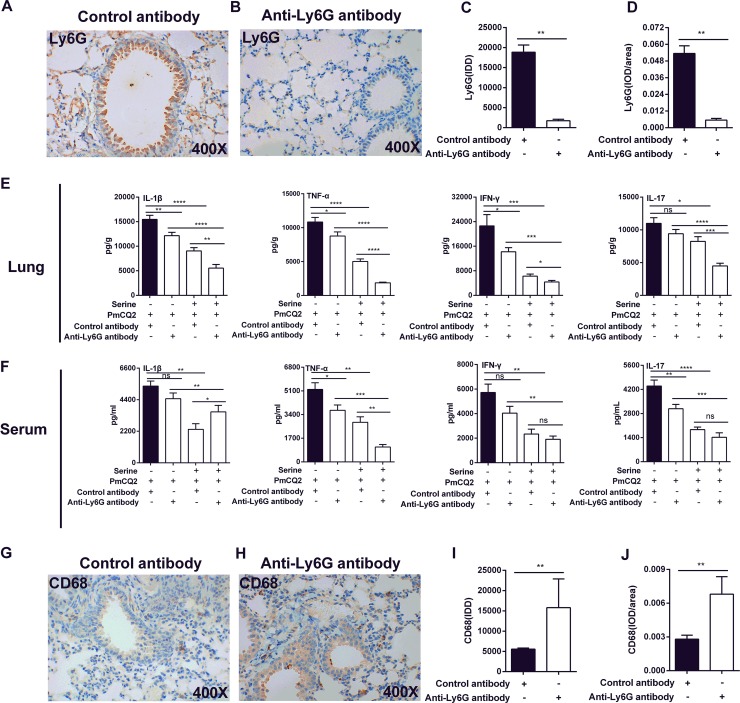
The role of l-serine in an alveolar neutrophil-cleared mouse model of P. multocida infection. Mice were infected with P. multocida by intraperitoneal injection, and the lung tissues were collected at 16 h after bacterial infection. (A to D) The expression of Ly6G was determined by immunohistochemistry (*n* = 6). (E and F) The production of IL-1β, IL-17, IFN-γ, and TNF-α in the lung and serum at 16 h after P. multocida infection (*n* = 8). (G to J) The expression of CD68 was determined by immunohistochemistry (*n* = 6). The data in panels C, D, I, and J were determined by unpaired *t* test or Mann-Whitney test, and panels E and F were analyzed by one-way ANOVA. All data were expressed as means ± SEM. *, *P* < 0.05; **, *P* < 0.01; ***, *P* < 0.001; ****, *P* < 0.0001.

To verify whether l-serine administration alleviates inflammation through modulating the functions of neutrophils, anti-Ly6G monoclonal antibodies were then used to eliminate neutrophils. Neutrophil elimination was confirmed by immunoblotting for Ly6G (Fig. 4A to D). Similar to the observations in macrophage-deleted models, serine supplementation significantly decreased the secretions of IL-1β, IL-17, IFN-γ, and TNF-α in the lung (Fig. 4E) and serum (Fig. 4F). Interestingly, the numbers of macrophages markedly increased after neutrophil elimination (Fig. 4G to J).

**FIG 5:**
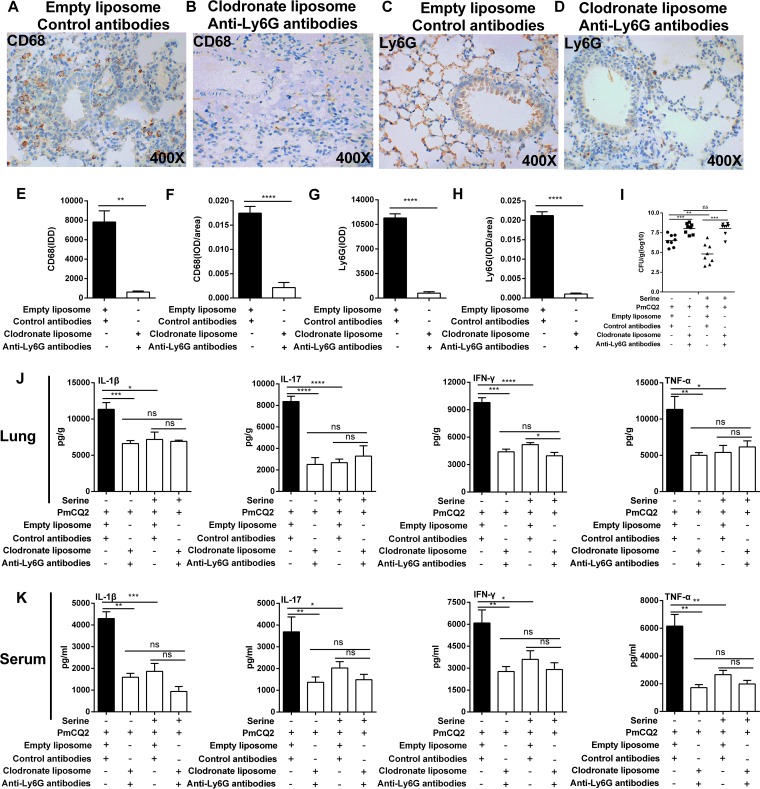
Serine reduces macrophage- and neutrophil-mediated inflammation during P. multocida infection. Mice were infected with P. multocida by intraperitoneal injection, and the lung tissues were collected at 16 h after bacterial infection. (A, B, E, and F) The expression of CD68 was determined by immunohistochemistry (*n* = 6). (C, D, G, and H) The expression of Ly6G was determined by immunohistochemistry (*n* = 6). (I) The bacterial burden in mouse lungs at 16 h after P. multocida infection (*n* = 10). (J and K) The production of IL-1β, IL-17, IFN-γ, and TNF-α in the lung and serum at 16 h after P. multocida infection (*n* = 8). The data in panels E, F, H, and I were analyzed by unpaired *t* test or Mann-Whitney test, and panels J and K were determined by one-way ANOVA. All data were expressed as means ± SEM. *, *P* < 0.05; **, *P* < 0.01; ***, *P* < 0.001; ****, *P* < 0.0001.

Thus, clodronate-loaded liposome and anti-Ly6G monoclonal antibody were used simultaneously to eliminate macrophages and neutrophils. Our results showed that about 90% of macrophages and neutrophils were eliminated (Fig. 5A to H). Although serine supplementation lowered the bacterial colonization in the lung without treatment of clodronate-loaded liposome and anti-Ly6G monoclonal antibody, serine supplementation failed to influence bacterial colonization in the lung (Fig. 5I) in the macrophage- and neutrophil-cleared model. More importantly, serine supplementation failed to affect the secretion of inflammatory cytokines in the lung (Fig. 5J) and serum (Fig. 5K) in the macrophage- and neutrophil-cleared model. Taken together, we conclude that l-serine reduces inflammatory responses mediated by macrophages and neutrophils during P. multocida infection.

## DISCUSSION

Knowledge about amino acid metabolic cross talk between a pathogen and its host has advanced in recent years and includes the following: (i) the host has the ability to alter amino acid metabolism after an infection, (ii) amino acids affect host immune responses against a pathogen, and (iii) amino acids play an important role in the physiology and virulence of pathogens (25). There are significant changes about the levels of amino acids during P. multocida infection. Notably, we found that P. multocida infection shapes the serine, glycine, and threonine metabolism and lowers the levels of glycine, threonine, and serine. This raises an interesting question about the underlying mechanism for downregulated l-serine during P. multocida infection. We have shown that the enzymes related to l-serine metabolism are significantly upregulated during P. multocida infection. Apart from the possibility of an increase in serine metabolism, whether the synthesis and transportation of l-serine is blocked during P. multocida infection remains to be further investigated. Besides serine, it is worth exploring the metabolic talk between P. multocida and the host for other amino acids. Indeed, previous studies have found that exogenous addition of arginine, glutamine, and/or proline highly shapes the pathogenesis of P. multocida infection (17–19).

In addition to their nutritional functions, amino acids also play important roles in the modulation of the inflammatory responses during bacterial infection (26). In this study, serine inhibits macrophage- and neutrophil-mediated inflammatory responses. Besides macrophages and neutrophils, other immune cells are also involved in the pathogenesis of P. multocida infection, like effector T cells (9), dendritic cells (27), and even B cells (28). Thus, it is interesting to know whether serine affects the functions of other immune cells, such as T cells, B cells, and dendritic cells, during P. multocida infection. We have found that depletion of macrophages and neutrophils increases the bacterial burden in the lung, and this depletion cannot fully block the cytokine production; thus, it is interesting to uncover the influence of serine on functions of other immune cells, especially Th1 cells and Th17 cells, during P. multocida infection.

Macrophages are the first line of defense against host pathogen invasion (29). Notably, inflammasomes, including NALP1, NLRP3, NLRC4, and AIM2, are essential for regulating the inflammatory responses in macrophages (30) by activating the proinflammatory protease caspase-1 to promote the maturation and secretion of IL-1β (31–33). IKK/NF-κB signaling pathways are involved in the regulation of the inflammatory responses (34), and NF-κB is a key transcriptional regulator of the macrophages (35). Mammalian target of rapamycin (mTOR) is a central metabolic pathway that couples nutrient sensing to the regulation of metabolic processes and affects macrophage polarization (36, 37). It is interesting to explore the underlying mechanisms by which serine inhibits the macrophage polarization, like the activation of inflammasome, NF-κB, and mTOR signaling.

It is well known that neutrophils have the same important roles as macrophages in the participation of inflammatory responses (38–40). Although macrophages and neutrophils have phagocytic and bactericidal functions (41–45), the main function of neutrophils is nonspecific defense (innate immunity) (46), while macrophages are involved in innate immunity and specific defense (cellular immunity) (47). Furthermore, in macrophages, enhanced pro-IL-1β processing is dependent on caspase-1; however, in neutrophils, the secretion of IL-1β is dependent on serine proteases (48–50). In this study, the results showed that l-serine has immunosuppressive effects on macrophages and neutrophils; however, whether the underlying mechanism for the immunosuppressive effect of serine on neutrophils is consistent with that in macrophages still needs to be revealed.

Another interesting observation is that l-serine reduces the colonization of P. multocida in mice. Notably, serine does not directly affect the growth and adhesion of P. multocida. A possible explanation is that serine inhibits the production of inflammatory cytokines, resulting in a lower bacterial load of P. multocida. A previous study has found that the bacterial load of Listeria monocytogenes in the mouse liver is associated with the production of inflammatory cytokines (e.g., IL-18), and administration of IL-18 promotes the load of L. monocytogenes (51). Another study found that ablating inflammatory monocytes or impairing their recruitment to the lungs improves murine survival and reduces fungal proliferation and dissemination (52). Similarly, a study found that clearance of neutrophils enhances mouse survival and reduces bacterial colonization and inflammation induced by Ehrlichia chaffeensis (53).

This experiment is based on a mouse model; however, the P. multocida strain used is mainly isolated from cattle and mainly causes infection in cattle. Therefore, further experimental exploration is needed, including whether serine is resistant to infection by other sources (e.g., poultry and pig) or different serotype strains (e.g., B, D, E, and F) and whether serine has anti-infective effects on different hosts (e.g., bovine, birds, or human).

In conclusion, we found that l-serine levels in the lungs of mice infected with P. multocida are significantly downregulated. Notably, exogenous l-serine administration lowers bacterial colonization and macrophage- and neutrophil-mediated inflammation and enhances the survival rate in mice infected by P. multocida. Based on this study, l-serine can be considered a nutrient additive for the prevention of macrophage-associated diseases (e.g., inflammation, bacterial infection, and tissue damage) in animals and/or humans.

## MATERIALS AND METHODS

### Bacterial strains.

Bovine Pasteurella multocida serotype A strain CQ2 (PmCQ2) (GenBank accession number LIUN00000000) is a highly virulent strain (intramuscular 50% lethal dose [LD_50_] = 2.2 × 10^5^ CFU in mice) (10), which is isolated from a bovine lung. PmCQ2 was generally grown in Martin’s broth agar containing 5% horse serum at 37°C for 24 h (17).

### Mice.

Female C57BL/6 mice (weight, 18 to 22 g; 6 to 8 weeks old) were purchased from the Laboratory Animal Center of Third Military Medical University (Chongqing, China) and housed in individually ventilated, pathogen-free cages (temperatures of 20 to 30°C, relative humidity at 50 to 60%, lighting cycle at 12 h/day) with free access to food and water. This study was carried out in accordance with the principles of the Basel Declaration and recommendations of the Laboratory Animal Ethical Commission of Southwest University (permit number 11-1025), Chongqing, China.

### P. multocida infection in mice.

Mice were infected by an intraperitoneal injection with P. multocida at a dose of 2.2 × 10^5^ CFU (LD_50_) in 100 μl. In the control group, mice (gender and age matched) were injected intraperitoneally with an equal dose of saline. A total of 556 mice were used in this study. Survival rates (*n* =10) were measured in both groups after injection. Mice were also euthanized for collection of tissues and serum samples at 4 h (*n* = 6), 8 h (*n* = 6), and 16 h (*n* = 10) postinfection.

### Bacterial colonization.

To measure the bacterial load, the lung tissues of mice (*n* = 6 or 10) were collected at different time points after bacterial infection. The tissues were homogenized aseptically, and bacterial loads were quantified by 10-fold serial dilution in saline. These different dilutions were plated in triplicate on Martin’s broth agar and were incubated at 37°C up to 24 h to count CFU.

### Quantitative real-time PCR.

The lung tissue was quickly collected and stored in liquid nitrogen. Total RNA of the lungs was acquired as described previously (54). cDNA was synthesized using a PrimeScript RT reagent kit with genomic DNA (gDNA) eraser (TaKaRa, Dalian, China). Specific primers are listed in Table S2 in the supplemental material. Quantitative real-time PCR (qRT-PCR) was performed according to a previous study (9).

### Enzyme-linked immunosorbent assay.

Lung homogenates were frozen and thawed (frozen in liquid nitrogen for 5 min and then melted on ice) three times. After centrifugation at 12,000 rpm for 10 min at 4°C, supernatant was acquired. Cytokines (e.g., IL-17, IL-6, and IFN-γ) were detected in the supernatant or the serum with enzyme-linked immunosorbent assay (ELISA) kits in accordance with the manufacturer’s protocol. ELISA kits for cytokines were purchased from eBioscience (USA).

### Lung amino acid analysis.

Lung amino acids were analyzed with isotope dilution liquid chromatography-mass spectrometry methods as previously described (55).

### Clearance of alveolar macrophages in mice.

Macrophage clearance was assessed by referring to the method described in previous literature (56, 57). Briefly, the mice were completely anesthetized intraperitoneally with 80 μl of 1.5% pentobarbital sodium (Beijing Huaye Haoyu Chemical Co., Ltd.). Then, 200 ml of empty liposomes or liposome chlorophosphate (LIPOSOMA) was administered through the trachea into the mouse lung.

### Deletion of neutrophils in mice.

Neutrophil clearance was conducted based on the method described in the previous literature (58). Briefly, mice were treated with 300 mg anti-mouse Ly6G (clone1A8) antibodies (Bio X Cell) by intraperitoneal injection. Antibodies were administered every 2 days up to 4 times.

### Histopathological examination and immune-histochemical staining of tissue section.

The histopathological examination experiments were performed as described in a previous study (9). The immune-histochemical (IHC) experiment was conducted as described in a previous study (55). The fixed slices were dehydrated in graded ethanol, embedded in paraffin, sectioned, and stained with hematoxylin and eosin (H&E) for histopathological examinations. For the lung immunohistochemical staining of CD68 and Ly6G, deparaffinized lung sections were treated with 3% H_2_O_2_ in methanol for 30 min to block endogenous peroxidase, and tissue sections were boiled in 0.01 M citrate buffer for antigen retrieval and then blocked with 1% normal goat serum (SouthernBiotech, AL, USA) for 30 min at room temperature. Sections were then incubated overnight at 4°C with anti-CD68 antibody (1:500 dilution; Proteintech, China) or anti-Ly6G antibody (1:50 dilution; Abcam, United Kingdom). After washing in phosphate-buffered saline (PBS), the sections were visualized by biotinylated secondary antibodies followed by incubation with horseradish peroxidase (HRP)-conjugated streptavidin for 30 min (R&D Systems, London, UK). Then, sections were incubated with 3,3′-diaminobenzidine (DAB) (Aladdin, Shanghai, China) for 3 min. After washing in PBS, all specimens were lightly counterstained with hematoxylin for 5 min. The areas of CD68-positive (CD68^+^) or Ly6G-positive (Ly6G^+^) lung cells, as well as total areas of lung sections, were measured using Image-Pro Plus 6.0 software (Media Cybernetics, Inc., Rockville, MD, USA). CD68^+^ or Ly6G^+^ cells were examined in a total of 10 fields at ×400 magnification per animal. The data collectors were unaware of the treatment status of the examined slides.

### Phagocytosis and adhesion assay of macrophage and lung epithelial cells.

Peritoneal macrophages were isolated from mice as previously described (24, 59). Peritoneal macrophages and lung epithelial cells were cultured in RPMI 1640 medium (Gibco, USA) with 10% heat-inactivated fetal bovine serum (FBS) (Gibco, USA) and counted with a hemocytometer, and then incubated overnight at 37°C with 5% CO_2_ in 48-well microplates at a density of 2 × 10^5^ cells/well. Then cells were washed with PBS to remove the nonadherent cells. The adherent cells were cultured in RPMI 1640 medium supplemented with 10 mM l-serine, threonine, or glycine for 2 h and subsequently challenged with P. multocida at a multiplicity of infection (MOI) of 1 for 16 h. Cells were washed three times with chilled PBS to remove nonassociated bacteria and lysed in PBS containing 0.1% Triton X-100. The cell lysates were diluted with PBS and grown on Martin’s agar plates at 37°C for 18 to 24 h to determine the number of P. multocida cells (total number of adhered and phagocytosed P. multocida cells). The cells of another 48-well microplate were treated with ciprofloxacin (100 mg/ml) for 30 min and washed 3 times with PBS to remove extracellular ciprofloxacin. Then cells were lysed by addition of 0.1% (vol/vol) Triton X-100 in PBS to count numbers of P. multocida cells (number of phagocytosed P. multocida cells) (11). The number of adhered P. multocida cells was equal to the total number of adhered and phagocytosed P. multocida cells minus the number of phagocytosed P. multocida cells.

### Statistical analyses.

Data shown are the means ± the standard error of the mean (SEM). Data were statistically analyzed according to our previous papers (60, 61). Data between two groups were analyzed by unpaired *t* test (Prism 6.0) if the data were in Gaussian distribution and had equal variance, by unpaired *t* test with Welch’s correction (Prism 6.0) if the data were in Gaussian distribution but showed unequal variance, or by nonparametric test (Mann-Whitney U test, Prism 6.0) if the data were not normally distributed. The Gaussian distribution of data was analyzed by the D’Agostino-Pearson omnibus normality test (Prism 6.0) and the Kolmogorov-Smirnov test (Prism 6.0). The variance of data was analyzed by the Brown-Forsythe test (Prism 6.0). Differences with a *P* value of <0.05 were considered significant.

## Supplementary Material

Supplemental file 1

Supplemental file 2

Supplemental file 3
