# Supplementary material for: l-Serine Lowers the Inflammatory Responses during Pasteurella multocida Infection
Source: Infect Immun. 2019 Nov 18;87(12):e00677-19. doi: 10.1128/IAI.00677-19 (PMC6867830; doi:10.1128/IAI.00677-19)
Supplement: Supplemental file 3 [file IAI.00677-19-s0003.pdf]

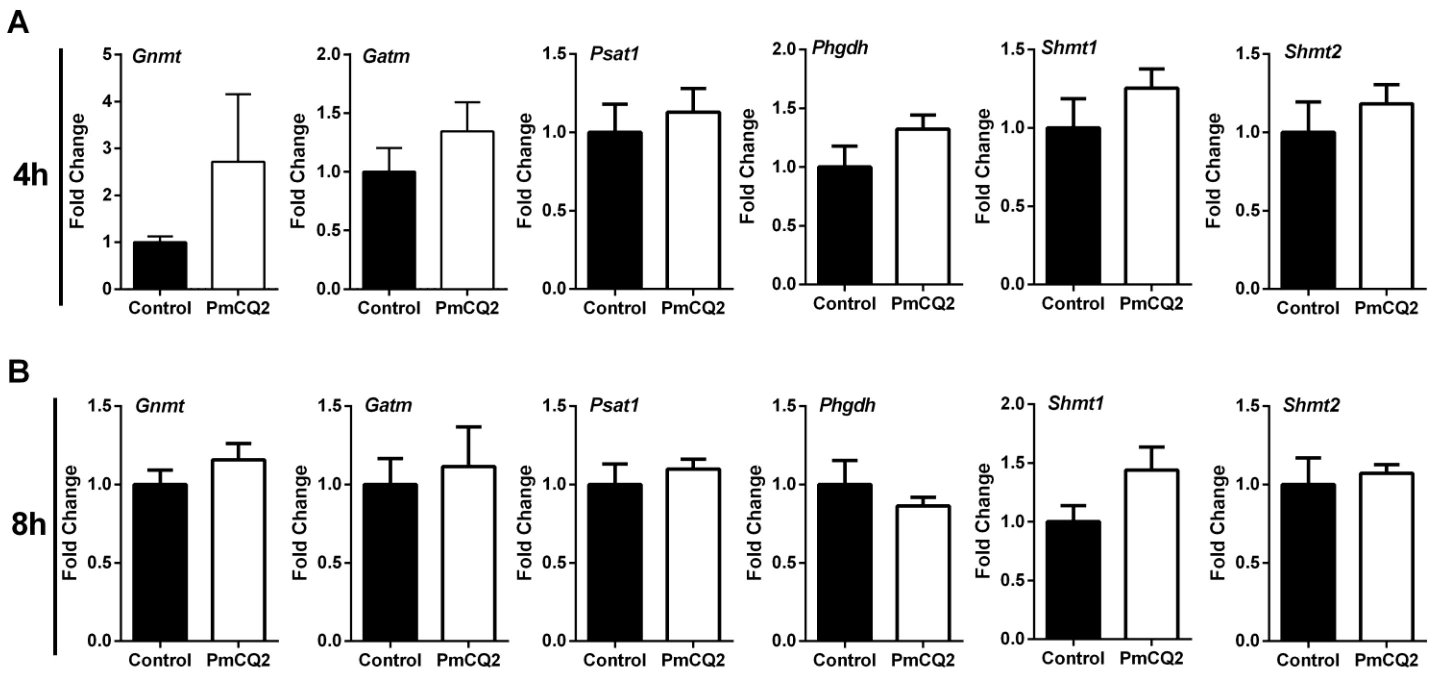

FIG S1 Gene expression of L-serine metabolism-related enzymes after *P. multocida* infection. **(A)** Gene expression of L-serine metabolism-related enzymes at 4h post infection (n=6). **(B)** Gene expression of L-serine metabolism-related enzymes at 8h post infection (n=6). The data were analyzed with unpaired t test or Mann-Whitney test and were expressed as means ± SEM.

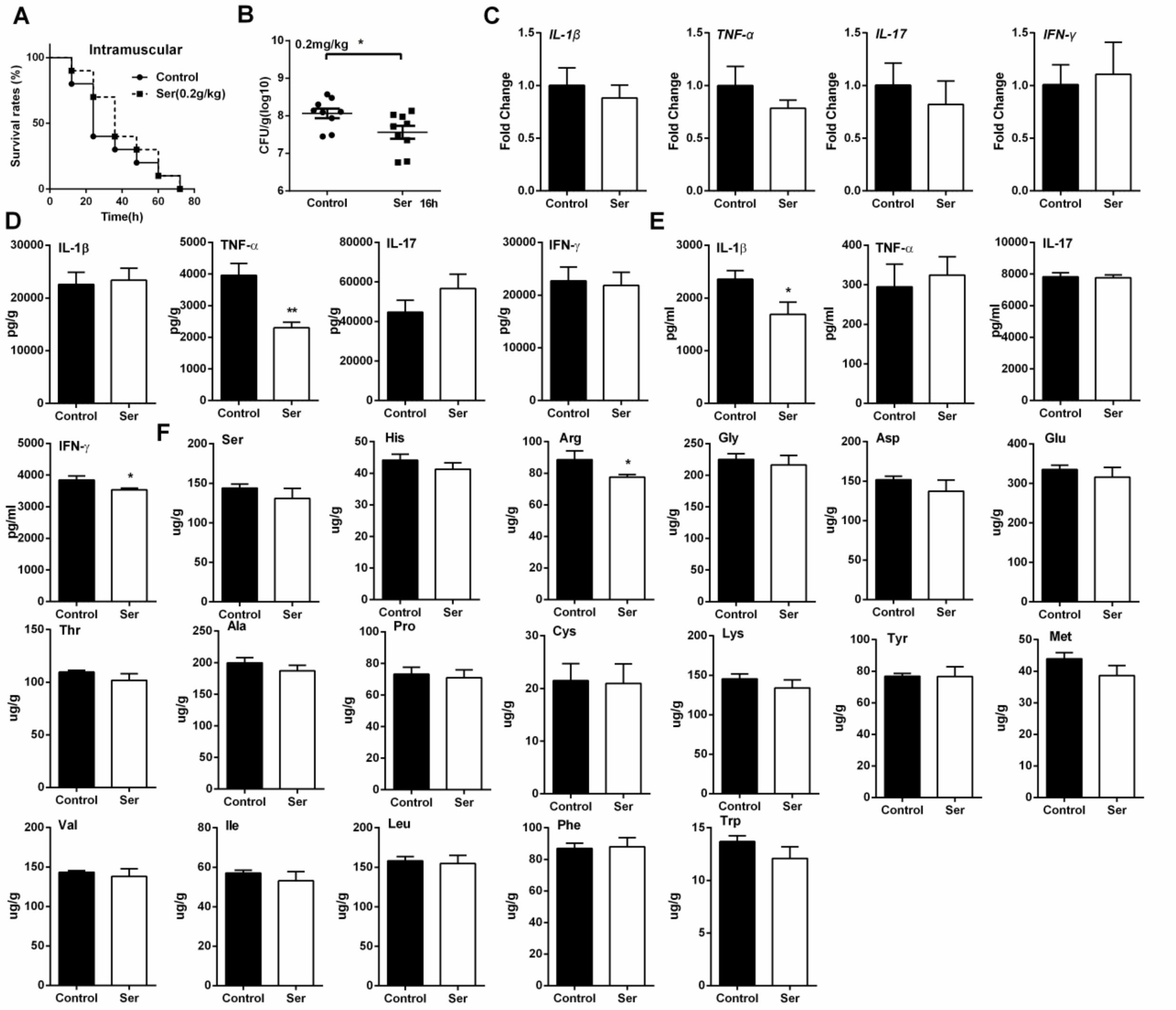

FIG S2 The effect of exogenous addition of L-serine via intramuscular injection on the mice infected by *P. multocida* infection. **(A)** The survival rate of mice by *P. multocida* infection after exogenous L-serine (0.2g/kg) addition via intramuscular injection (n=10). **(B)** The colonization of *P. multocida* in the lung at 16h post infection after exogenous L-serine (0.2g/kg) addition via intramuscular injection (n=9). **(C)** The expression of inflammatory factors after exogenous addition of L-serine (0.2g/kg) in lung tissues at 16h post infection by *P. multocida* (n=9). **(D-E)** The production of inflammatory cytokines after exogenous addition of L-serine (0.2g/kg) in lung tissues and serum at 16h post infection by *P. multocida* (n=9). (F) Exogenous addition 0.2g/kg L-serine did not affect the levels of amino acids in mice lungs at 16h post *P. multocida* infection (n=9). Data were analyzed by unpaired t-test or Mann-Whitney test and shown as the means ± SEM. *P < 0.05.

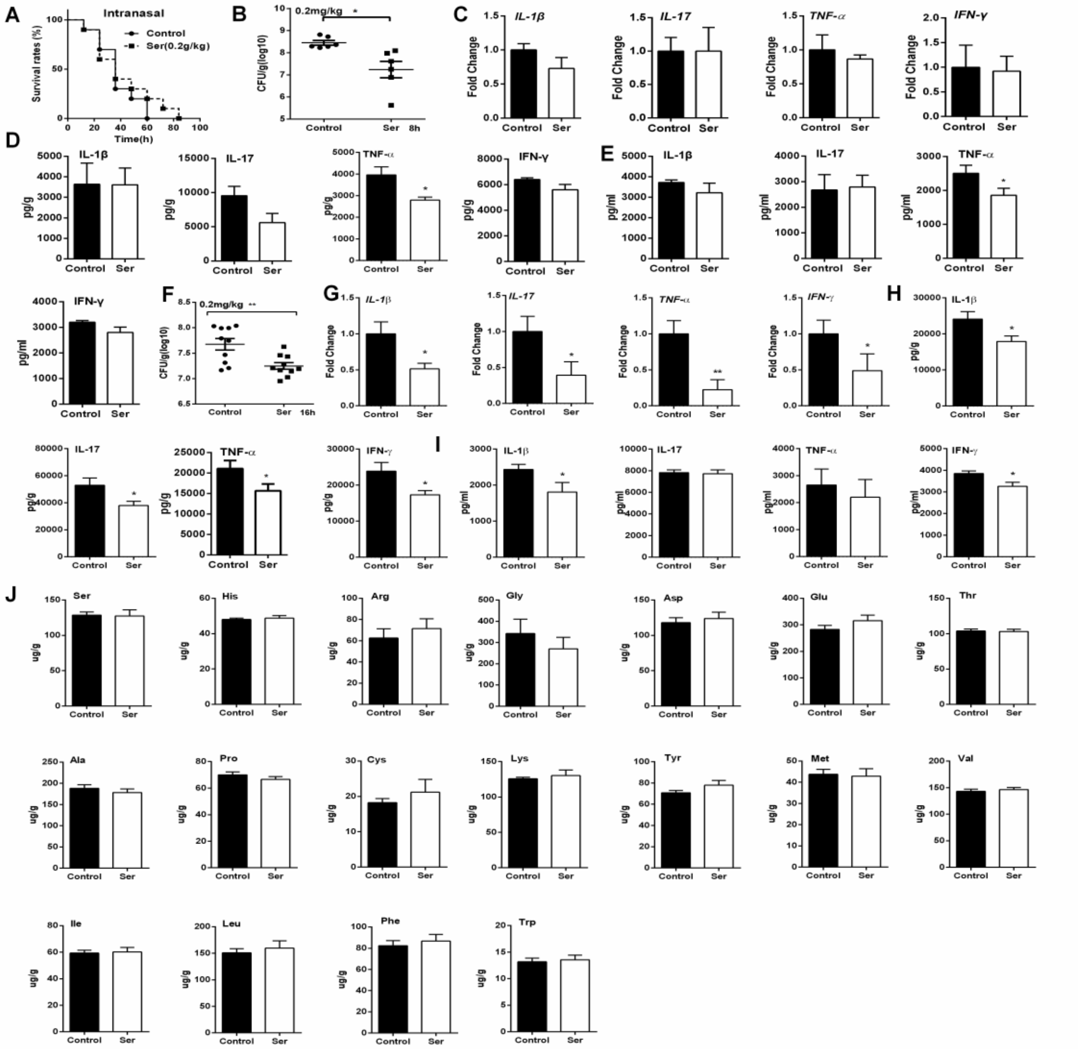

FIG S3 Effects of intranasal injection of 0.2g/kg L-serine on mice infected by *P. multocida*. **(A)** The survival rate of mice infected by *P. multocida* (n=10). **(B)** The bacterial colonization in the lungs of mice at 8h post *P. multocida* infection (n=6) **(C)** The expression of inflammatory factors through qRT-PCR at 8h post *P. multocida* infection (n=6). **(D-E)** The production of inflammatory cytokines in lung tissues and serum at 8h post infection by *P. multocida* (n=6). **(F)** The bacterial colonization in the lungs of mice at 16h post *P. multocida* infection (n=10). **(G)** The expression of inflammatory factors at 16h post *P. multocida* infection (n=10). **(H-I)** The production of inflammatory cytokines in lung tissues and serum at 16h post infection by *P. multocida* (n=10). **(J)** Exogenous addition 0.2g/kg L-serine via intranasal injection does not affect the levels of amino acids in the lung tissues of mice by *P. multocida* at 16h post infection (n=6). **(A)**, **(B)** and **(F)** were representative of three independent experiments (n = 6-10 total mice per group). **(C)-(E)** and **(G)-(I)** were pooled from two independent experiments (n = 6-10 total mice per group). Data were analyzed by unpaired t-test or Mann-Whitney test and expressed as means means ± SEM. $^{*}P < 0.05$, $^{**}P < 0.01$.

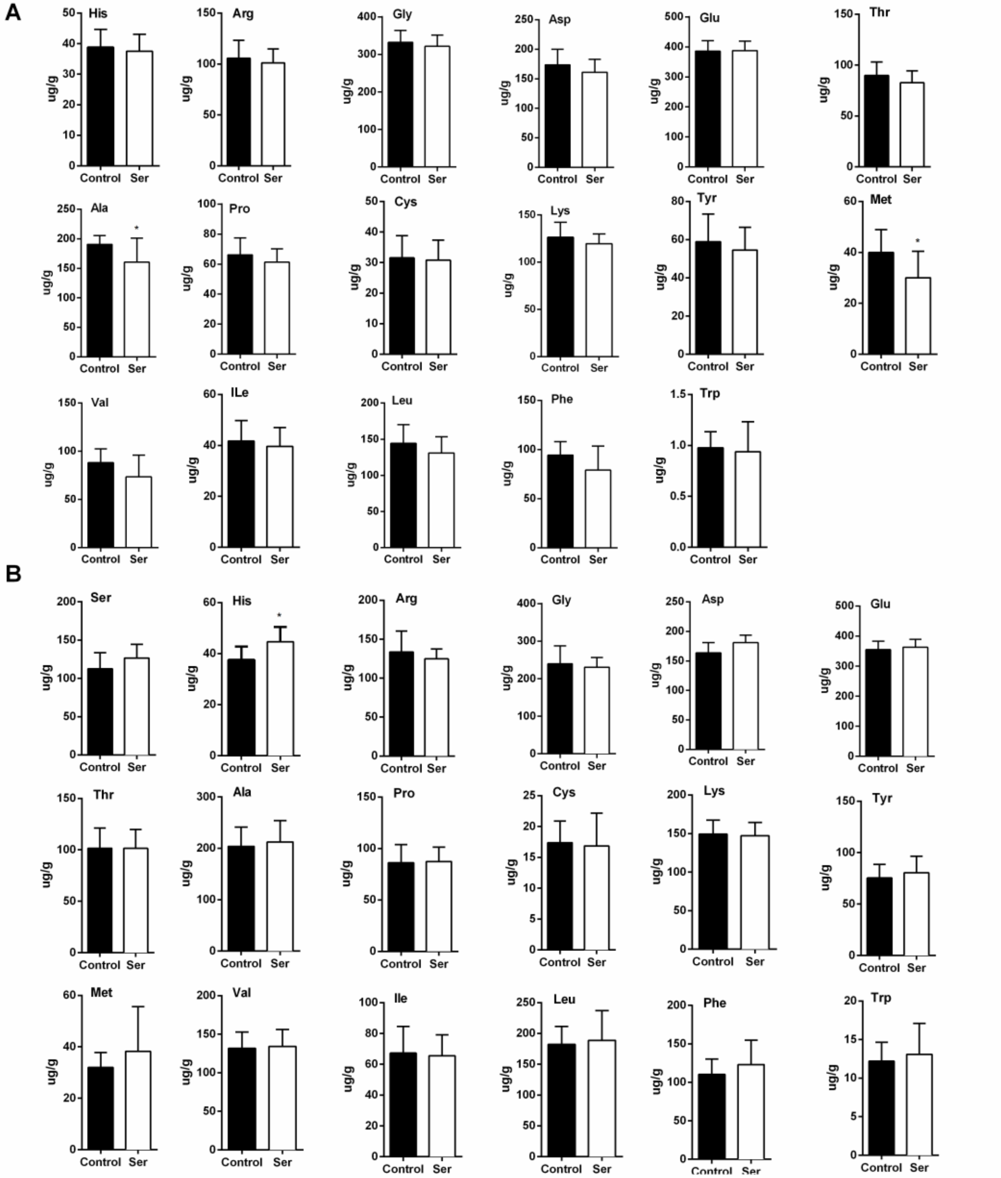

FIG S4 The levels of amino acids in the lungs of mice by *P. multocida* after exogenously adding of L-serine (2g/kg) at 8h and 16h post infection. **(A)** The concentration of amino acids in lung tissues of mice after adding of L-serine (2mg/kg) by intranasal injection at 8h post *P. multocida* infection (n=10). **(B)** The concentration of amino acids in lung tissues of mice after adding of L-serine (2mg/kg) by intranasal injection at 16h post *P. multocida* infection (n=6). The data were analyzed with unpaired t test and expressed as means ± SEM. *P < 0.05.

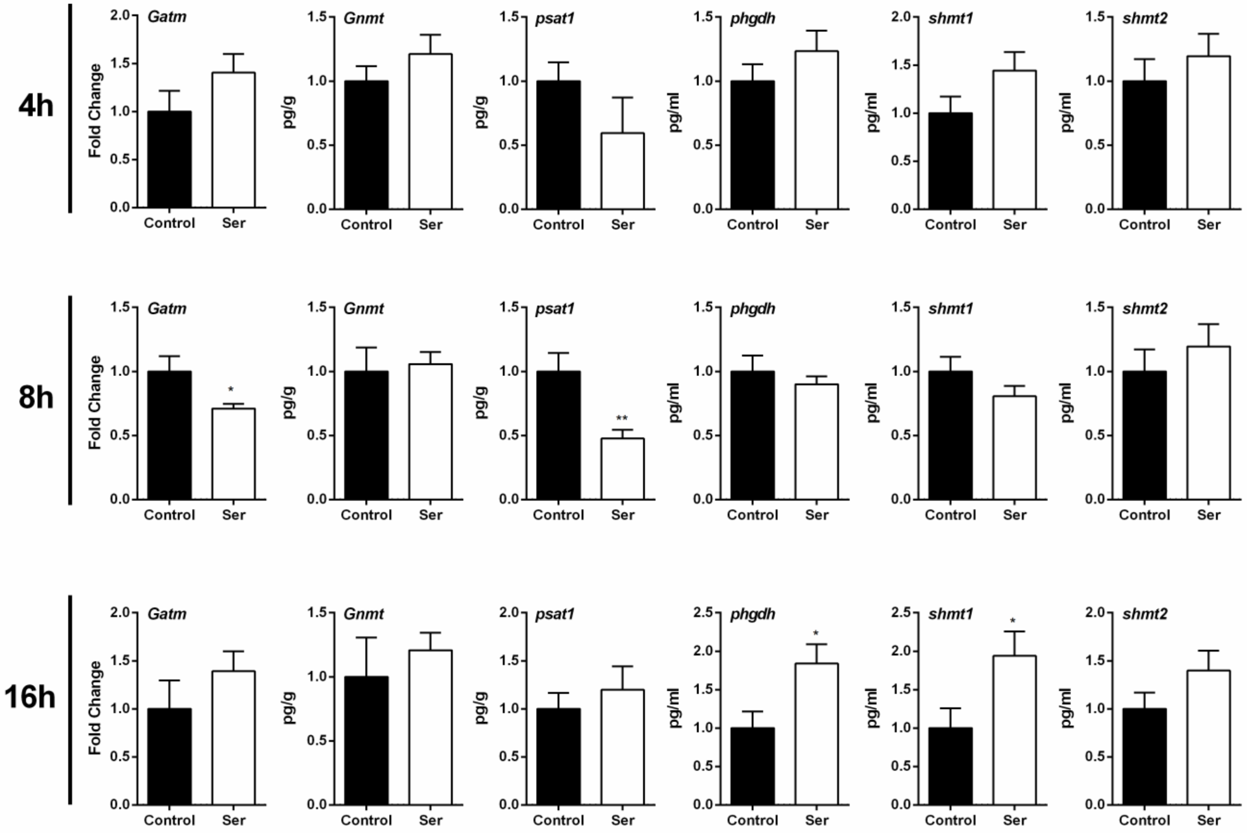

FIG S5 Expression of L-serine metabolism-related enzymes in the lungs of mice at 4h, 8h and 16h post infection by *P. multocida* after exogenously adding of L-serine (2g/kg, n = 6-10). The data were analyzed with unpaired t test and shown as means ± SEM. $^*P < 0.05$.

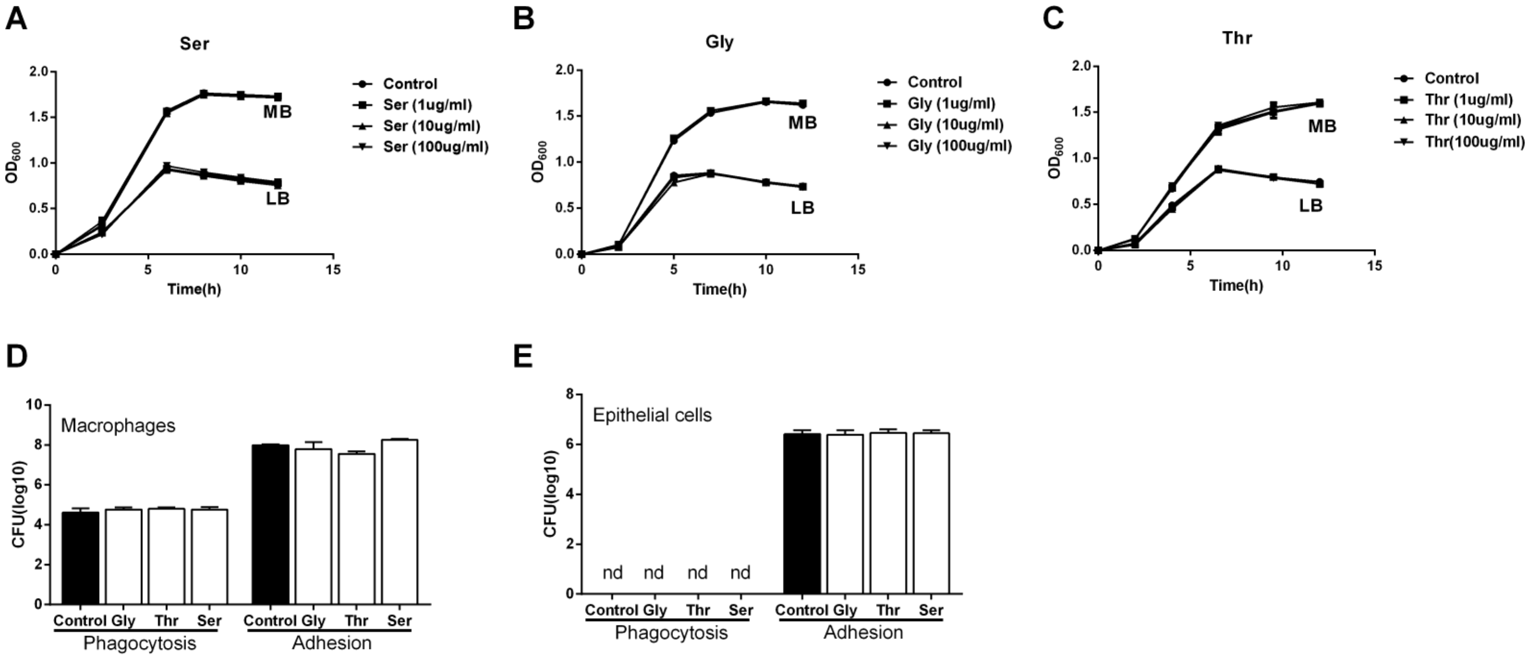

FIG S6 The effect of glycine, threonine and serine on the growth and adherence of *P. multocida*. The growth of *P. multocida* was analyzed after serine **(A)**, glycine **(B)** and threonine **(C)** supplementation (n=3). The adherence of *P. multocida* to macrophages **(D)** and epithelial cells **(E)** were analyzed after serine, glycine and threonine supplementation (n=6).**(A)**-**(C)** are representative of three independent experiments with 8 replicates in each time, **(D)** and **(E)** were pooled from two independent experiments (n = 6). **(A)**, **(B)**, and **(C)** was determined with unpaired t test, **(D)** and **(E)** were analyzed with one-way ANOVA, and data were expressed as means ± SEM.
